# Specific enhancement of the translation of thermospermine-responsive uORF-containing mRNAs by ribosomal mutations in *Arabidopsis thaliana*

**DOI:** 10.1080/15592324.2025.2480231

**Published:** 2025-03-15

**Authors:** Koki Mutsuda, Yuichi Nishii, Tomohiko Toyoshima, Hiroko Fukushima, Hiroyasu Motose, Taku Takahashi

**Affiliations:** Graduate School of Environmental, Life, Natural Science and Technology, Okayama University, Okayama, Japan

**Keywords:** mRNA translation, RPL10, suppressor mutant, thermospermine, uORF

## Abstract

Auxin-induced xylem formation in angiosperms is negatively regulated by thermospermine, whose biosynthesis is also induced by auxin. In *Arabidopsis thaliana*, loss-of-function mutants of *ACL5*, which encodes thermospermine synthase, exhibit a dwarf phenotype accompanied by excessive xylem formation. Studies of suppressor mutants that recover from the *acl5* dwarf phenotype suggest that thermospermine alleviates the inhibitory effect of an upstream open-reading frame (uORF) on the main ORF translation of *SAC51* mRNA. Many suppressor mutations for *acl5* have been mapped to the uORF conserved in the *SAC51* family or to ribosomal protein genes, such as *RPL10A*, *RPL4A*, and *RACK1A*. In this study, we identified newly isolated *acl5* suppressors, *sac501*, *sac504*, and *sac506*, which are additional alleles of *RPL10A* and the uORFs of *SAC51* family members, *SACL1* and *SACL3*, respectively. To investigate whether *acl5*-suppressor alleles of ribosomal genes broadly affect translation of uORF-containing mRNAs, we examined GUS activity in several 5’-GUS fusion constructs. Our results showed that these alleles enhanced GUS activity in *SAC51* and *SACL3* 5’-fusion constructs but had no effect on other 5’-fusion constructs unrelated to thermospermine response. This suggests that these ribosomal proteins are specifically involved in the thermospermine-mediated regulation of mRNA translation.

## Introduction

Polyamines play a role in various aspects of plant growth and development.^1–3^ In angiosperms, in addition to the essential role of spermidine conserved across eukaryotes in the hypusine modification of a translation factor eIF5α, putrescine and spermine, whose biosynthesis is upregulated by ABA, serve as polycations to help retain water in the cell under salinity or drought stress. Spermine can also be a source of hydrogen peroxide in biotic defense responses.^4^ On the other hand, its structural isomer, thermospermine, is specifically produced in vascular tissues and involved in negative regulation of xylem formation.^5^ Loss-of-function mutants of the *ACAULIS5* (*ACL5*) gene in *Arabidopsis thaliana*, which encodes thermospermine synthase, show increased xylem vessel formation and a dwarf phenotype. Expression of the *ACL5* gene is induced by auxin, which triggers vascular patterning, but is reduced by thermospermine, indicating that both auxin-induced xylem formation and themospermine biosynthesis are under negative feedback control by thermospermine.^6^ Studies of suppressor mutants that recover the dwarf phenotype of *acl5* without thermospermine have revealed that thermospermine enhances the mRNA translation of *SAC51*, which encodes a basic loop-helix-loop (bHLH) protein involved in repressing xylem formation, by alleviating the inhibitory effect of an upstream open-reading-frame (uORF) on the main ORF of the *SAC51* mRNA.^7^ As *acl5* suppressors, we have so far identified dominant or semidominant alleles of the uORF of *SAC51* and *SACL3*, a member of the *SAC51* family,^7–9^ as well as alleles of ribosomal protein genes, including *RPL4A*, *RPL10A*, and *RACK1A*,^10,11^ and a recessive allele of *JMJ22*, which encodes a Jumonji C (JmjC) domain-containing protein of the JMJD6 family.^12^ Although these mutations may affect mRNA translation or stability of the *SAC51* family, their precise mechanisms in substituting for the action of thermospermine remain unclear. Here, we identify the genes responsible for additional *acl5* suppressor mutants, *sac501-d*, *sac504-d*, and *sac506-d*, and further show that the ribosomal mutations that suppress *acl5* preferentially affect the mRNA translation of the *SAC51* family.

## Materials and methods

*sac501-d*, *sac504-d*, and *sac506-d* were identified from ethyl methanesulfonate (EMS)-mutagenized seeds of the original *acl5–1* in the Landsberg *erecta* (L*er*) accession of *Arabidopsis thaliana*,^11^ and crossed with Columbia-0 (Col-0) more than five times to characterize in the Col-0 background. *sac52-d*, *sac53-d*, *sac56-d*, and T-DNA insertion mutants, *sac51–1*, *sacl1–1*, *sacl2–1*, and *sacl3–1* were described previously.^9–11^

Map-based cloning of the genes responsible for *sac501-d*, *sac504-d*, and *sac504-d* was performed as described previously.^11,12^ Whole genome sequencing of these mutants was conducted using next-generation sequencing technology on the MGI DNBSEQ-G400 system at the Bioengineering Lab (Sagamihara, Japan). Sequence information for the *sac501-d* allele of *RPL10A* and the PCR primers used to detect this genotype during the generation of multiple mutants are shown in Supplementary Figure S1.

The T-DNA construct, consisting of the CaMV 35S promoter and the *SAC51* 5’ leader-β-glucuronidase (GUS) fusion gene, is as described previously.^11^ Other 5’ leader-GUS fusions were made by inserting each 5’ genomic fragment, amplified by PCR, between the 35S promoter and the GUS reporter gene of pBI121 (Clontech, USA). PCR primers used are shown in Supplementary Table S1. T-DNA constructs were introduced into the wild-type Col-0 genome by *Agrobacterium*-mediated transformation,^13^ and the resulting transgenic lines were selected by growth on kanamycin-containing medium. GUS activity was measured according to the standard protocol.^14^

## Results and discussion

**Figure 1.**
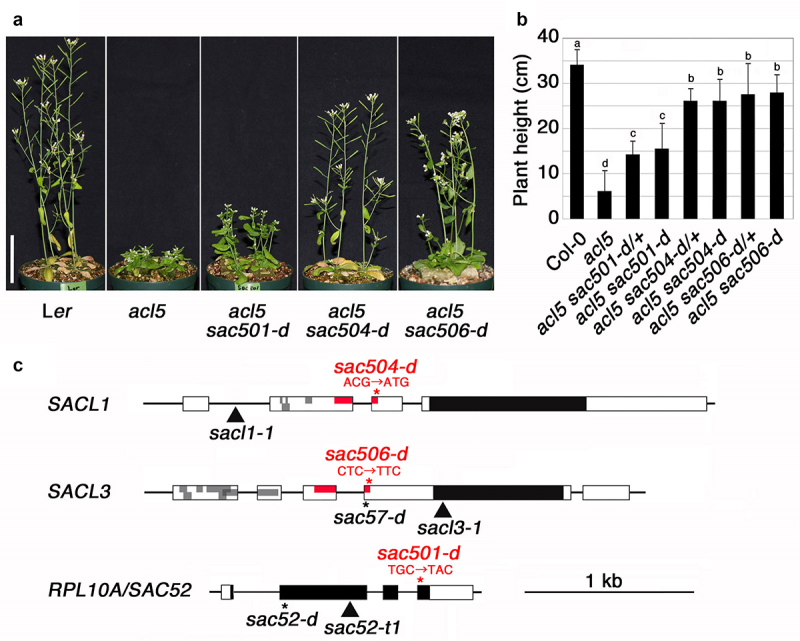
Identification of *sac501-d*, *sac504-d*, and *sac506-d*. (a) Growth phenotype of 40-day-old wild-type L*er*, *acl5–1*, and each suppressor mutant in the *acl5–1* background grown under 16 h light/8 h dark conditions. Bar = 5 cm. (b) Plant height comparison of 40-day-old plants in the col-0 background. “/+” indicates a heterozygote with the wild-type allele. Error bars represent the SD (*n* = 10). Different letters indicate significant differences at the 0.05 level using ANOVA. (c) Genomic structure of *SACL1*, *SACL3*, and *RPL10A/SAC52*. Boxes indicate exons. Gray and black areas represent uORFs and a main ORF, respectively. Asterisks indicate positions of base substitutions in each allele. The alleles identified in this study are highlighted in red letters with codon changes. Arrowheads indicate T-DNA insertion alleles.

*sac501*, *sac504*, and *sac506*, which were isolated as suppressor mutants for *acl5*,^11^ exhibit partial but significant recovery of plant height compared to *acl5*, although the degrees of recovery differ among them (Figure 1a). These mutants were found to be dominant and are referred to as the *d* alleles because there was no morphological difference between the homozygous mutant plants in the *acl5* background and their F1 plants from crosses with *acl5* (Figure 1b). Mapping and whole genome sequencing revealed that *sac501-d*, *sac504-d*, and *sac506-d* harbor point mutations that cause amino acid substitutions in *RPL10A* and the conserved uORFs of *SACL1* and *SACL3*, respectively (Figure 1c). We have previously identified another mutant of *RPL10A* and a mutant of the uORF of *SACL3* as suppressors of *acl5*, namely *sac52-d* and *sac57-d*.^9,10^ Different alleles of the uORFs of *SACL1* and *SACL3* have also been isolated through similar screening for suppressor mutants of *acl5*.^15^ All four members of the *SAC51* family contain multiple uORFs and the suppressor alleles identified so far have point mutations in the uORFs, whose sequences are highly conserved among family members and have an inhibitory effect on the translation of the main ORF.^16^ These results suggest that overproduction of SAC51, SACL1 or SACL3 bHLH proteins is sufficient to suppress the *acl5* phenotype. Incidentally, we confirmed that each single mutant of *sac501-d*, *sac504-d*, and *sac506-d* does not exhibit any morphological abnormalities.

**Figure 2.**
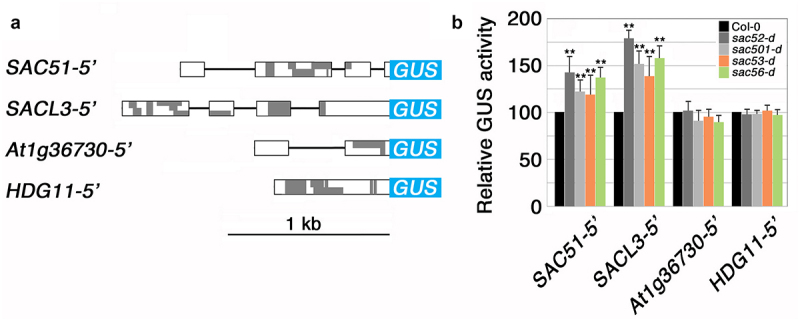
Effect of the ribosomal mutations that suppress *acl5* on the GUS activity from the 35S–5’-GUS fusions. (a) The GUS fusion constructs consisting of the CaMV 35S promoter, the 5’ leader region of the genes tested, and the GUS reporter. Boxes, bars, and gray areas indicate exons, introns, and uOrfs, respectively. (b) Relative GUS activity in the mutants carrying each GUS construct. A representative transgenic line for each construct was crossed with each ribosomal mutant to make plants homozygous for both the mutation and the GUS gene. Samples were prepared from 7-day-old seedlings grown on MS agar plates. Error bars represent the SD (*n* = 5). Asterisks indicate the significant differences from wild-type (Student’s *t*-test, **p < 0.01).

Our previous study using the *SAC51* 5’-GUS fusion gene revealed that *acl5*-suppressor alleles of ribosomal protein genes *RPL10A*, *RPL4A*, and *RACK1A*, named *sac52-d*, *sac53-d*, and *sac56-d*, respectively, enhance the translation of the *SAC51* main ORF.^11^ Here, we examined whether these ribosomal mutations, including *sac501-d*, generally affect the translation of uORF-containing mRNAs using transgenic lines carrying 5’-GUS genes under the control of the CaMV 35S promoter (Figure 2a). The GUS activity from *SAC51* 5’-GUS and *SACL3* 5’-GUS constructs was significantly higher in *sac52-d*, *sac53-d*, *sac56-d* and *sac501-d* compared to the wild-type background (Figure 2b). On the other hand, GUS activity from *At1g36730* 5’-GUS and *HDG11* 5’-GUS fusions was not increased by these ribosomal mutations. The *At1g3*6730 5’ leader contains a uORF that is conserved among homologous genes across species and is annotated as CPuORF19.^17,18^ This uORF has been shown to respond to water limitation.^19^ *HDG11*, which encodes a homeodomain protein of the class IV HD-Zip family,^20^ contains multiple uORFs in its 5’ leader, and their peptide sequences may not be conserved among different plant species. We used *HDG11* as a representative of a random uORF-containing gene. Our results suggest that the mutations in *RPL10A*, *RPL4A*, and *RACK1A* that suppress *acl5* preferentially affect the conserved uORFs of the *SAC51* family, whose effect on main ORF translation is derepressed by thermospermine. However, it is still likely that there are other mRNAs, both with and without uORFs, whose translation is modified by these mutations. Regarding *sac53-d*, a mutant of *RACK1A*, the single mutant shows growth defects, including slight dwarfism and shortened hypocotyls in etiolated seedlings.^11^

**Figure 3.**
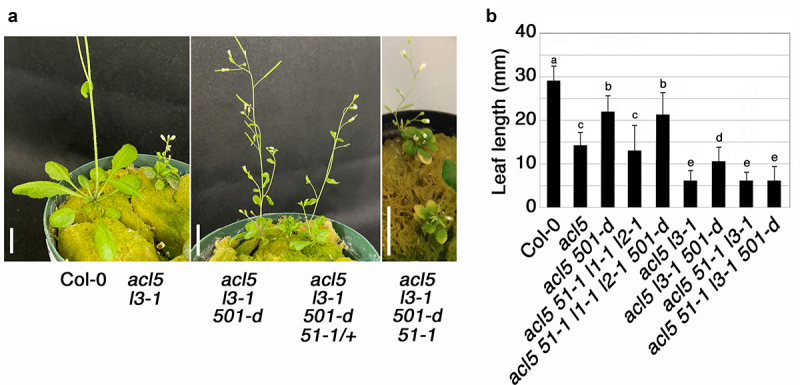
Requirement for *SAC51* and *SACL3* in the suppression of *acl5* by *sac501-d*. (a) Effect of *sac501-d* on the tiny plant phenotype of *acl5–1 sacl3–1*. Morphology of 35-day-old col-0 and each mutant plant is shown. Bars = 1 cm. (b) Leaf length comparison among col-0 and each mutant. The 5th and 6th leaves of 35-day-old plants were taken for measurement. Error bars represent the SD (*n* = 10). Different letters indicate significant differences at the 0.05 level using ANOVA. The notation “*sac*” is omitted from mutant names.

We then examined whether the suppression of the *acl5* phenotype by *sac501-d* is attributable to enhanced translation of *SAC51* and *SACL3* mRNAs, and hence, the function of these proteins. The *acl5* phenotype is exacerbated by a knockout allele of *SACL3*, *sacl3–1*, and the *acl5 sacl3–1* mutant has very tiny inflorescences and leaves.^9^ A triple mutant of *acl5 sacl3–1 sac501-d* remarkably suppressed the tiny plant phenotype of *acl5 sacl3–1* (Figure 3a). However, the tiny plant phenotype of *acl5 sacl3–1 sac51–1*, which is identical to that of *acl5 sacl3–1*, was not suppressed by *sac501-d* (Figure 3a), suggesting that *SAC51* is required for this suppression. On the other hand, the *acl5* phenotype is not altered by knockout alleles of other *SAC51* family members, *sac51–1*, *sacl1–1*, and *sacl2–1*. The dwarf phenotype of a quadruple mutant, *acl5 sac51–1 sacl1–1 sacl2–1*, was suppressed by *sac501-d* to a similar extent as in *acl5 sac501-d*, as shown by the leaf length in Figure 3b. These results suggest the importance of *SAC51* and *SACL3* in the suppression of *acl5* by *sac501-d* and likely other ribosomal mutations.

In conclusion, our study of suppressor mutants for *acl5* emphasizes the key role of uORF-mediated mRNA translation of the *SAC51* family, particularly *SAC51* and *SACL3*, in thermospermine-dependent plant growth. Translation of these mRNAs may be preferentially enhanced by the ribosomal mutations identified so far as *acl5*-suppressor alleles. Given that cellular polyamines generally interact with RNAs, it is possible that thermospermine is structurally integrated into ribosomes by interacting with rRNAs in xylem precursor cells, where thermospermine is present.

## Supplementary Material

FigureS1.docx

TableS1.docx
