# Supplementary material for: Specific enhancement of the translation of thermospermine-responsive uORF-containing mRNAs by ribosomal mutations in Arabidopsis thaliana
Source: Plant Signal Behav. 2025 Mar 15;20(1):2480231. doi: 10.1080/15592324.2025.2480231 (PMC11913374; doi:10.1080/15592324.2025.2480231)
Supplement: FigureS1.docx [file KPSB_A_2480231_SM5472.docx]

**Supplementary Figure S1.**

(A) A partial genomic sequence of *RPL10A/UL16Z/At1g14320*. Protein coding sequences are boxed. A point mutation in *sac501-d* is shown in red. The dCAPS primers for detecting *sac501-d* are shown in bold and italic letters.

gacttttttttcagGGGCTTCACGAAGTTTAACAGAGCTGACTTCACCAAGTTGAGGCAAGAGAAGCGTGTTGTCCCTGATGGTGTCAACGCTAAGgtattattggctttacatcagaattgtcactctttgatcctatagcagtgatagttatcattggaaatatcatttgttgttgaattgaacaat***gttttcgtttttttttcagTTCCTC***T***CAT*G**CCATGGACCTTTGGCTAACCGTCAGCCGGGAAGTGCCTTTTTGCCAGCCCACTACTGAagagtatcagaactgaagtatccttctcattccggtgaagaagaattataatcagcctgaatctttttacttatcgttatctctggtgttgttttaagtttttagttggacacaatcagtattctgaatctttttgtgactcttttgtttaagctctgaaatgattttgttccttcgttcttggccatatatctttcatttgcaagttttatcatggctttagctttaaatattttaattgaagatttgcttgaaaatcgttgaagttttag***atagagatgggtgattgcct***tgtt

(B) The dCAPS primer sequences and the lengths of DNA fragments amplified and *Sph*I-digested.

501dCAPS-F: gttttcgtttttttttcagTTCCTCGCAT

501dCAPS-R: AGGCAATCACCCATCTCTAT

Wt: 350bp+30bp, *sac501-d*: 380bp
