# Supplementary material for: Specific enhancement of the translation of thermospermine-responsive uORF-containing mRNAs by ribosomal mutations in Arabidopsis thaliana
Source: Plant Signal Behav. 2025 Mar 15;20(1):2480231. doi: 10.1080/15592324.2025.2480231 (PMC11913374; doi:10.1080/15592324.2025.2480231)
Supplement: TableS1.docx [file KPSB_A_2480231_SM5471.docx]

**Supplementary Table S1.** Primer sequences for the 5’-GUS fusion construction.

*Spe*I*, Xba*I, *Bgl*II or *Bam*HI restriction sequences added to be inserted into the *Xba*I-*Bam*HI sites of pBI121 are shown in bold letters.

| Gene name | Gene ID | Forward primer | Reverse primer |
| --- | --- | --- | --- |
| *SACL3* | At1g29950 | **ACTAGT**TTATCAGATGATTGCTGA | **AGATCT**AAAAAAGGAAACGCAAAGAG |
| - | At1g36730 | **ACTAGT**cttctcatttctagtttctcag | **GGATCc**gtaaacaaacaagggatgtcag |
| *HDG11* | At1g73360 | **TCTAGA**AGAAGGTTGGTTCGGTCCT | **GGATCC**CTTGAAACTCCTGTCCAAG |
